# Design of Lightweight CFRP Automotive Part as an Alternative for Steel Part by Thickness and Lay-Up Optimization

**DOI:** 10.3390/ma12142309

**Published:** 2019-07-19

**Authors:** Jeong-Min Lee, Byeong-Jin Min, Joon-Hong Park, Dong-Hwan Kim, Byung-Min Kim, Dae-Cheol Ko

**Affiliations:** 1 PNU-IFAM JRC, Pusan National University, 63, Busandaehak-ro, Geumjeong-gu, Busan 46241, Korea; 2 Graduate School of Convergence Science, Pusan National University, 63, Busandaehak-ro, Geumjeong-gu, Busan 46241, Korea; 3 Department of Mechanical Engineering, Dong-A University, Busan 49315, Korea; 4 Aircraft Parts Engineering, International University of Korea, Jinju-si 52833, Korea; 5 Precision Manufacturing Systems Division, Pusan National University, 63, Busandaehak-ro, Geumjeong-gu, Busan 46241, Korea

**Keywords:** carbon fiber reinforced plastic (CFRP), structural analysis, genetic algorithms (GAs), optimization, B-pillar, reinforcement, drop tower test

## Abstract

Mechanical properties, such as strength and stiffness, of laminated carbon fiber reinforced plastic (CFRP) are generally affected by the lay-up method. However, no precise design rules to replace steel products with CFRP have been established that satisfy these properties. Therefore, this study proposes a set of rules to design automotive parts with equivalent bending stiffness through structural analysis and genetic algorithms (GAs). First, the thickness of the CFRP product was determined by comparing the bending deformation of steel products by structural analysis. To minimize the orthotropic characteristics of CFRP, the quasi-isotropic lay-up method was implemented to determine the thickness. Next, the lay-up angle was determined using GAs. The optimized lay-up angle of the CFRP product with minimum bending deformation was determined by population generation, cross-over, mutation, and fitness evaluation. CFRP B-pillar reinforcement was fabricated using the determined conditions and the bending deformation of the single component was evaluated. Finally, the B-pillar assembled with CFRP reinforcement was investigated by the drop tower test.

## 1. Introduction

Carbon fiber reinforced plastic (CFRP) finds its use in various applications including aircraft, machinery, sports equipment, and automobile. Reducing the weight of automotive parts has lately become an important issue to solve environmental protection problems and reduce fuel consumption. Various attempts have been made to replace steel products with CFRP products, which have equivalent mechanical properties (strength and stiffness) [1,2,3,4].

Generally, a design rule that satisfies the equivalent mechanical properties of steel products must be implemented to the frames of CFRP automotive parts. Most studies conducted on the design rules of CFRP focused on predicting the strength and stiffness of simple shapes considering the weaving method of materials, thickness, and lay-up angle [5,6,7]. Soremekun [8] studied a lamination method using modified genetic algorithms (GAs). Design parameters such as the weaving method of materials, thickness, and lay-up angle were considered to minimize the weight and maximize the bending stiffness for the CFRP plate. However, designing complex shapes, such as automotive parts, using CFRP is difficult because the bending stiffness of simple shapes was calculated by the classical lamination theory (CLT). To implement complex shape composites in automotive parts, Kim et al. [9] studied the design of composite bumper beam using impact analysis combined with micro-GAs. This study showed that the optimally designed composite bumper beam reduced the weight by 33% as compared to steel products and improved the impact performance. Similarly, Belingardi et al. [10] studied the design optimization of automotive bumpers using composite and recyclable thermoplastic materials, wherein the thickness of the CFRP product was determined considering the stiffness of the steel product. However, they did not study the design of lay-up angle to reinforce the bending stiffness of laminated CFRP. Several studies have been conducted on the design and optimization of CFRP products. However, the design procedure of the CFRP product that satisfies the mechanical properties of steel products with complex shape has not been established [11,12,13,14].

This study aims to propose the design rules of automotive parts for B-pillar reinforcement with bending stiffness equivalent to steel products. The designing of rules was classified into two stages: Determination of thickness and optimization of lay-up angle. First, the thickness of the product with the greatest effect on the bending stiffness was determined by structural analysis. In the first stage, the thickness was determined by adding one ply to the CFRP product until the required bending stiffness was achieved. The second stage optimized the lay-up angle of the CFRP product by implementing GAs. Because the thickness (number of plies) was determined in the first stage, GAs used the lay-up angle as a design parameter. The lay-up angle with maximum bending stiffness was determined through GAs and its bending stiffness was compared to that of steel products. Next, the validity of the design rules was evaluated by the bending test of CFRP reinforcement manufactured by implementing the designed thickness and lay-up angle. Finally, the drop tower test was performed using the assembled B-pillar to evaluate whether CFRP products can replace steel products.

## 2. Methodology

### 2.1. Material

A commercial twill weave prepreg (fabricated by SK Chemicals, Seongnam-si, Korea) was used in this study. A polyester resin based on thermoplastic polyurethane with a glass transition temperature (Tg) of 110 °C was used. The thickness of the prepreg was 0.3 mm. The carbon fiber volume fraction and density of the prepreg were evaluated to be 45% and 1.52 g/cm^2^, respectively.

To obtain the mechanical properties of cured CFRP, tensile tests were performed in accordance with ASTM D3039 [15]. The universal test machine (MTS, 10 ton, Eden Prairie, MN, USA) was used to conduct the test at a constant rate of 2 mm/min. The flat plates laid-up by [0°]_5_ were cut at 0°, 45°, and 90° with dimensions 250 × 25 × 1.5 mm^3^ to fabricate the tensile specimens. The results of the tensile tests with CFRP showed that E_11_ and E_22_ were 40.35 GPa, G_12_ was 9.51 GPa, (strain range 0.1–0.3%) and ν_12_ was 0.13. The mechanical properties of G_13_ and G_23_ in the other direction calculated by Hooke’s law are listed in Table 1.

### 2.2. Quasi-Isotropic Lamination Method

The thickness of the laminate was determined during designing the CFRP product. Owing to the excessive computation time, it is recommended that the thickness and lay-up angle should not be considered simultaneously. Therefore, the thickness is calculated considering the lay-up angle with the same mechanical properties according to the directions.

**Figure 1:**
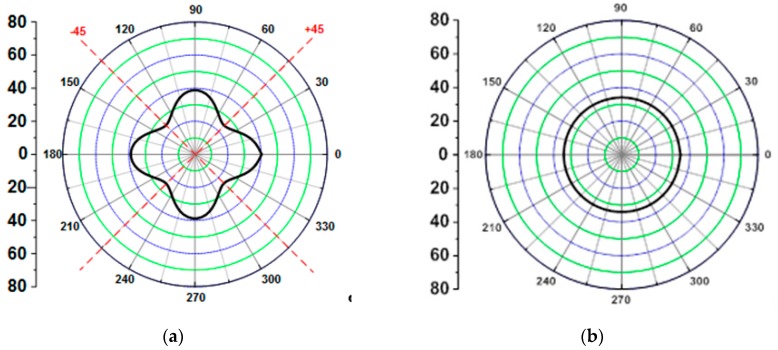
Polar diagram of elastic modulus for CFRP. (**a**) [0]_n_; (**b**) [0/45]_n_.

Generally, the elastic modulus of CFRP at 0°, 45°, and 90° was obtained by the tensile test, while Equation (1) was used to calculate its value at other angles [16].

(1) $$E_{\alpha \beta \left[\theta \right]}=\frac{1-\nu_{\alpha \beta }\nu_{\beta \alpha }}{\nu_{\alpha \beta }{Q_{\beta \alpha }}^{\left[\theta \right]}}$$

where E denotes the elastic modulus, *Q* is the stiffness matrix, *θ* is the lay-up angle of the layer, and *ν* is the Poisson’s ratio. The elastic modulus obtained with this equation can be used to represent the polar diagram. The elastic modulus at ±45° was much lesser than that at 0° and 90° where the laminate was laid-up to be [0]_n_, as shown in Figure 1a. If the load acts at ±45°, the thickness of the CFRP product may significantly increase. However, the lay-up angle calculated by the quasi-isotropic laminate method of [0/45]_n_ exhibits elastic modulus that is similar to the isotropic material, as shown in Figure 1b. In this study, the initial thickness of the CFRP product depended on the direction of the applied load because the lay-up angle was not considered in the first stage. Therefore, the quasi-isotropic lamination method was used to minimize the effect of orthotropic properties while determining the initial thickness [17,18,19,20].

### 2.3. Design Procedure of CFRP Product

**Figure 2:**
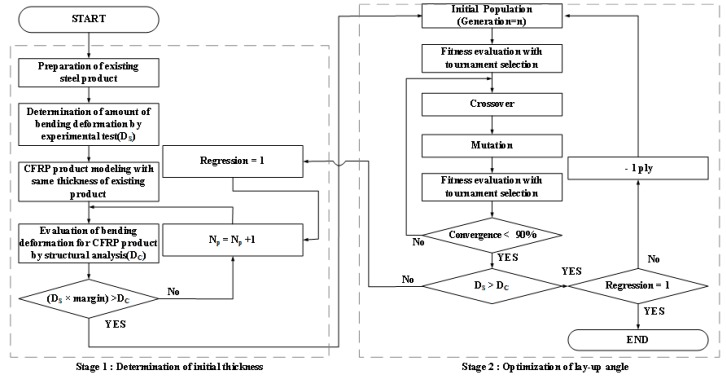
Flowchart of the design procedure for CFRP products.

In this study, the designing of rules was classified into two stages: Determination of initial thickness and optimization of lay-up angle to simplify the design of the CFRP product. Figure 2 shows the flowchart of the design procedure. In the first stage, the bending deformation (D_S_) of steel products was determined through structural analysis. The measurement of the bending stiffness of complex shaped products was difficult. Therefore, the amount of bending deformation applied at the load was compared [21,22,23]. Next, the CFRP product with thickness same as that of steel product was modeled and evaluated through structural analysis of linear finite element simulation. To reduce the difference in mechanical properties due to orthotropic characteristics of the CFRP product, the quasi-isotropic lamination method of [0/45]_n_ was implemented. The bending deformation of the CFRP product (D_C_) was then compared with that of steel products in the same position. The target value in the first stage was determined by adding 10% margin to the D_S_ because the second stage could strengthen D_C_. If the D_C_ did not satisfy the target value, a ply was added. This process was repeated until the target value was achieved. It is also one method to perform local reinforcement [24]. In this study, the simple method was applied to add a ply to the CFRP product considering manufacturing process.

In the second stage, the lay-up angle was optimized though structural analysis and GAs. First, the lay-up angle was determined considering the symmetry of the orthotropic material. The initial population was then generated by random selection. Second, the quality was evaluated through structural analysis. In addition, the tournament selection with the amount of minimum D_C_ for the CFRP product was applied to determine the next generation. Next, the crossover and mutation processes were conducted using the selected results. Finally, the quality and convergence of results were determined. When the convergence was more than 90%, GAs were terminated and the CFRP product with determined lay-up method by GAs was compared with the D_S_. If the target value was achieved, one ply was removed from the CFRP product and the second stage was re-executed to assess whether its thickness can be reduced. However, if the target value was not achieved, the second stage was performed by adding one more ply.

## 3. Application of Design Rules to B-Pillar Reinforcement

### 3.1. Determination of Thickness

**Figure 3:**
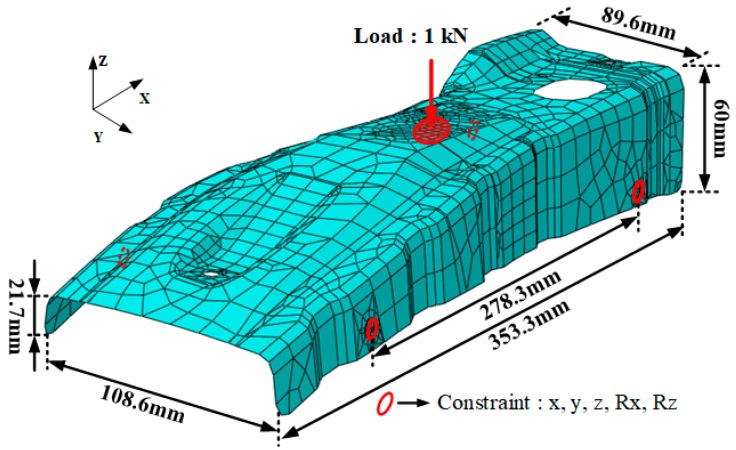
Structural analysis model of B-pillar reinforcement.

To determine the thickness of the B-pillar reinforcement, structural analysis was conducted by the commercial program ABAQUS 2019, where linear static analysis was used to reduce the computation time because several cases were analyzed in the GAs. A total of 3546 shell elements were used in the structural analysis of the B-pillar reinforcement model. As shown in Figure 3, constraint conditions were given to four areas fixed in x, y, and z-axes and rotation of x and z. A load of 1 kN was applied at the top of the product surface.

**Figure 4:**
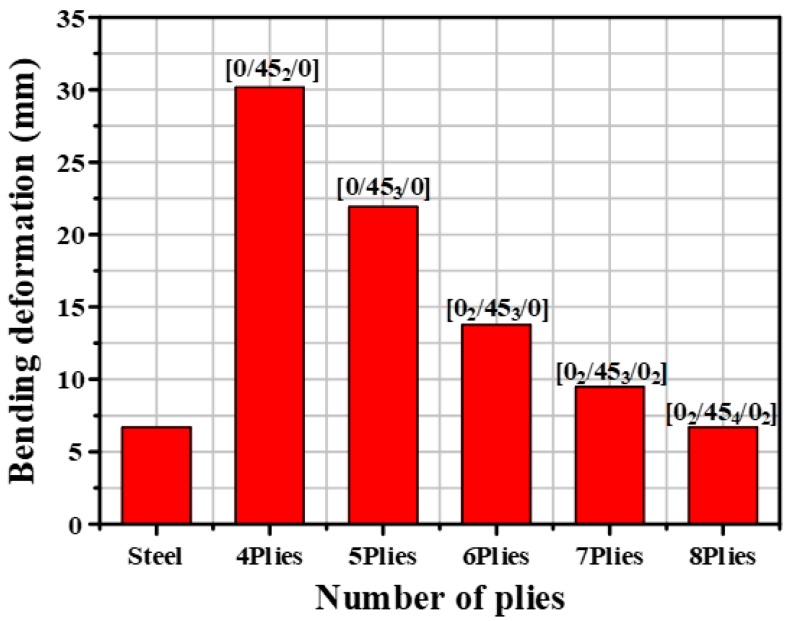
Results of bending analysis for CFRP B-pillar reinforcement.

Through structural analysis, the amount of D_S_ was calculated to be 2.26 mm. Therefore, the target value was 2.49 mm to consider the 10% margin of D_S_. Based on this result, structural analysis of the CFRP product was first performed at 1.2 mm thickness. Figure 4 shows the results of structural analysis for varying thickness by adding a ply to the CFRP B-pillar reinforcement until the target value was achieved. The amount of D_C_ with 1.2 mm thickness to stack 4 plies was 8.40 mm higher than the target value. The structural analysis of other thickness was conducted by adding one ply under same conditions. Consequently, the target value was achieved at thickness 2.4 mm to stack 8 plies. Therefore, the thickness of the CFRP B-pillar reinforcement was determined to be 2.4 mm.

### 3.2. Determination of Lay-Up Method Using GAs

In the second stage, GAs were used to obtain the optimized lay-up angle for the CFRP product. Table 2 shows the parameters of GAs to conduct the optimization of the lay-up angle for. Using the thickness 2.4 mm determined in Section 3.2, the lay-up angles of the laminate were determined to be 0°, 15°, 30°, 45°, 60°, and 75°, considering the symmetry of the orthotropic material. In addition, the minimum amount of D_C_ was considered the objective function of the quality evaluation.

**Figure 5:**
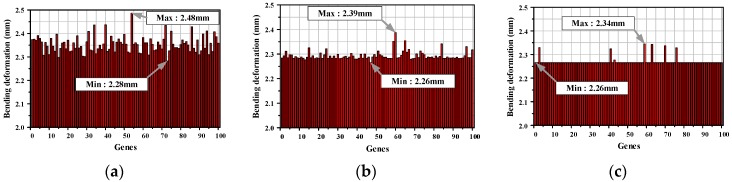
Results of GAs method for the optimization of fiber array. (**a**) 1st generation; (**b**) 5th generation; (**c**) 10th generation.

The procedure to optimize the lay-up angle of the laminate is as follows. First, the initial population was generated by the random selection method. The parameters of the lay-up angles 0°, 15°, 30°, 45°, 60°, and 75° were applied to each layer of the laminate consisting of eight plies. Second, the quality was evaluated by comparing the amount of D_C_ depending on the lay-up angle through structural analysis. The tournament selection method was employed to generate the next 20% of the population. For tournament selection, two chromosomes were randomly selected and the better quality was transferred to the next generation. Third, the crossover operator was used to interchange and combine the genes between individuals. The one-point crossover method was employed to generate 70% of the offspring from random parents. Next, mutation was used to convert random genes to genetic diversity with 5% probability. Finally, steps 2–5 were repeated until the member of the population satisfied the stopping condition. In this study, the progress was stopped at the convergence of more than 90% of the population. The newly generated population with minimum D_C_ in the 5th generation showed more than 95% convergence at the 10th generation, as shown in Figure 5. Thus, the lay-up angle of converged population was evaluated to be [45°_2_/0°_3_/45°_2_/0°].

To verify the optimal lay-up angle of CFRP reinforcement, the amount of D_C_ was compared with that of steel products. The amount of D_C_ for the optimized CFRP product was 2.25 mm, which was satisfied by the bending deformation of 2.26 mm of steel products. Finally, one ply was removed from the CFRP product and the second stage was executed again to ensure the reduction in weight of the CFRP product. The amount of bending deformation of the CFRP reinforcement with seven plies (2.1 mm) was 2.81 mm, which did not satisfy the amount of D_S_. Therefore, the optimization of lay-up angle was determined to be [45°_2_/0°_3_/45°_2_/0°].

## 4. Verification of CFRP B-Pillar Reinforcement

### 4.1. Manufacturing of CFRP B-Pillar Reinforcement

**Figure 6:**
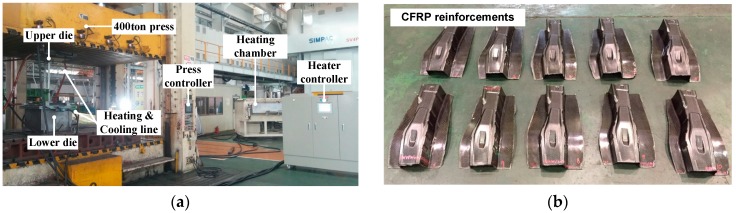
Experimental equipment and CFRP B-pillar reinforcements. (**a**) Manufacturing equipment; (**b**) CFRP reinforcements.

CFRP B-pillar reinforcements with the quasi-isotropic and optimal laminate methods were manufactured by the prepreg compression molding (PCM) process [25]. Figure 6a shows the experimental equipment consisting of the press, molds, heating and cooling system, and heating chamber to fabricate the CFRP B-pillar reinforcement. First, the pre-consolidated laminate that consisted of eight layers was cut into a dimension of 430 × 350 mm^2^ using a water-jet. To prevent the laminate from sticking to the mold surface, a liquid release agent for high temperature was spread on the mold surface. Second, the laminate was preheated to a temperature of 200 °C to prevent the convective heat loss below Tg when the laminate was transferred from the heating chamber to the mold. The heated laminate was transferred and placed on the forming mold heated to 150 °C. The laminate immediately deformed and the molds were then cooled down to 80 °C by the cooling water to demold the CFRP product. The manufactured B-pillar reinforcement is shown in Figure 6b. The final products were cut by the ultrasonic cutting machine to the same size as steel products.

### 4.2. Evaluation of CFRP B-Pillar Reinforcement

To determine weight reduction, the weights of the steel products and CFRP product were measured to be 540 and 200 g, respectively. Thus, a 62.96% reduction in the weight of B-pillar reinforcement was achieved by applying the CFRP material.

**Figure 7:**
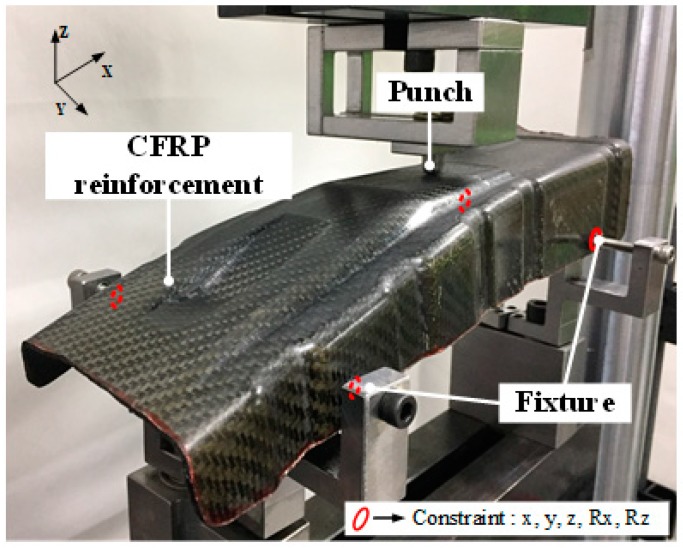
Experimental set-up for bending test.

Bending tests were performed to verify that the CFRP product satisfied the amount of D_S_. CFRP B-pillar reinforcements were fabricated by two lay-up angles [0°/45°_2_/0°] and [45°_2_/0°_3_/45°_2_/0°] and installed, as shown in Figure 7. The universal testing machine (MTS, 10 ton) was used to measure the stroke per load. The radius and load of the punch was 5 mm and 1 kN, respectively. The fixture frame used to install the CFRP B-pillar reinforcement was prepared to perform bending tests under the same conditions as those used in structural analysis.

**Figure 8:**
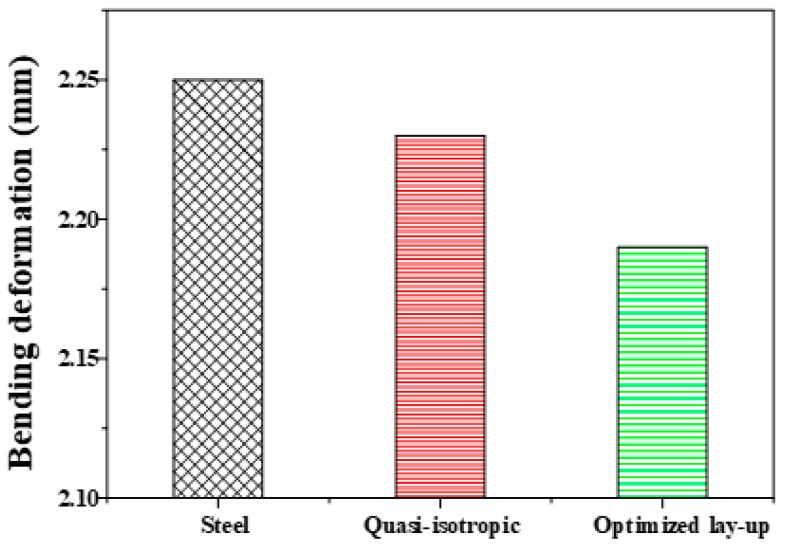
Experimental results of bending deformations for each product.

Figure 8 shows the experimental results of the bending deformation of B-pillar reinforcement made by steel, quasi-isotropic, and optimized lay-ups. The effectiveness of the design rules could be verified because the bending deformation of 2.19 mm of the optimized product was the smallest in comparison with steel products with bending deformation and quasi-isotropic product being 2.26 and 2.23 mm, respectively. However, the test results of bending deformation of both products were obviously reduced compared to the structural analysis. To assess this reason, forming analysis was performed in the same way as the manufacturing process using the mechanical properties of a previous study [26].

**Figure 9:**
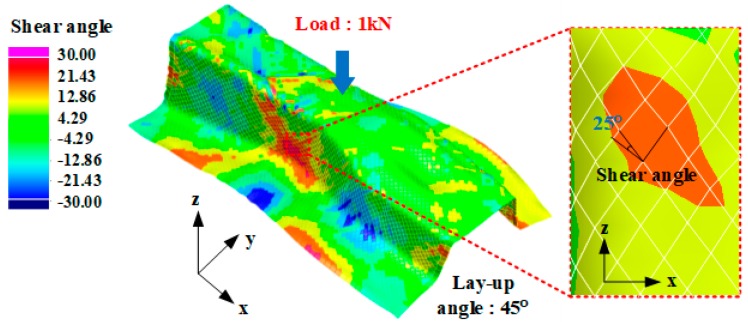
Result of forming analysis for CFRP reinforcement.

As shown in Figure 9, a shear angle occurred in the z-axis, where the bending load acted. Because the bending stiffness of the CFRP product was strengthened in the direction in which the fibers were arranged, bending deformation was smaller than structural analysis [27,28,29]. Therefore, structural analysis considering the shear angle that occurred in the product is required for precise product design.

**Figure 10:**
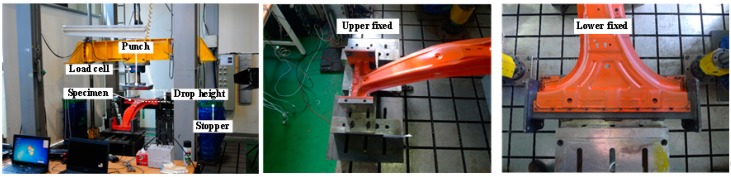
Equipment of drop tower test.

The drop tower test was performed using the B-pillar assembled with CFRP reinforcement to evaluate whether the designed product could achieve the same performance after assembly. The B-pillar and CFRP reinforcement were joined using adhesive bonding (TEROKAL 5055) made by HENKEL, Düsseldorf, Germany. Figure 10 shows the equipment of drop tower test to evaluate the energy absorption of B-pillar. The drop weight and height from the B-pillar were 400 kg and 500 mm, respectively. The B-pillar was welded using jigs fixed at the top and bottom in the direction of rotation considering the welding position of the automotive frame.

**Figure 11:**
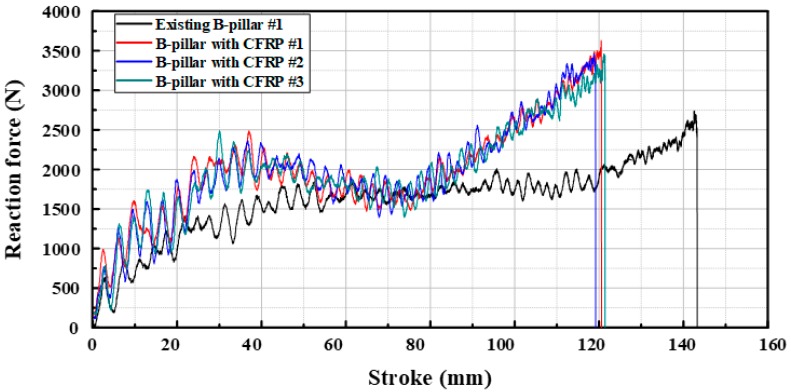
Results of drop tower tests.

**Figure 12:**
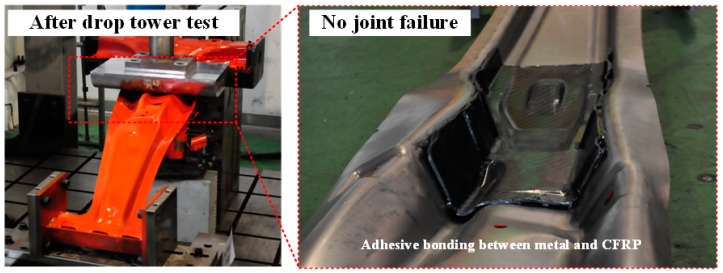
B-pillar with CFRP reinforcement after drop tower test.

Figure 11 shows the graph of the reaction force per stroke for the drop tower tests with the B-pillar (DP780) by applying DP590 and CFRP reinforcement. The experimental result shows that the reaction force of the B-pillar with CFRP reinforcement was higher in all strokes. In addition, this configuration had less strokes than the steel products. The energies absorbed by B-pillar with DP590 and CFRP were 2.252 kJ and 2.303 kJ, respectively. Both energy absorption and bending deformation were accurately evaluated for the B-pillar with CFRP reinforcement. The CFRP reinforcement was accurately attached to the B-pillar after the drop tower test and it was assumed that this adhesion performance prevented excessive bending deformation of the B-pillar, as shown in Figure 12. The conventional B-pillar was welded by the spot welding. The stiffness of conventional B-pillar was insufficient as comparted with the B-pillar applied CFRP in which the entire contact area was tied. Therefore, the design rules in this study are effective to replace the steel products with CFRP products.

## 5. Conclusions

In this study, the design rules of automotive parts with complex shape were proposed to replace the steel products with CFRP products that satisfies the mechanical properties such as strength and stiffness. The designing of rules was divided into two stages: Determination of thickness through structural analysis and determination of the lay-up angle through GAs with structural analysis. The design rules were validated by the bending test using the CFRP reinforcement, manufactured with the designed thickness and lay-up angle. Finally, the drop tower test was conducted using assembled B-pillar to determine whether CFRP products can replace steel products.

1. The thickness of CFRP product was determined by the quasi-isotropic laminate method to compare the bending deformation of steel products by structural analysis. Next, the lay-up angle was determined through structural analysis. The result of the lamination angle [45°_2_/0°_3_/45°_2_/0°] at 2.4 mm was obtained that satisfied the D_S_;
2. CFRP B-pillar reinforcement was fabricated as the determined conditions by PCM process. In order to evaluate weight reduction of B-pillar reinforcement, the weight was measured and compared to steel product with CFRP product. As a result, 62.96% weight reduction of B-pillar reinforcement was achieved in this study;
3. Bending test of single component was performed to compare the bending deformation of steel products with CFRP products. The effectiveness of the design rule was verified because the bending deformation (2.19 mm) of the optimized product was lower than that of steel products (2.25 mm);
4. Drop tower test was performed using the assembled B-pillar with CFRP reinforcement to evaluate whether the designed product could achieve the same performance after assembly. The experimental result shows that the reaction force of B-pillar with CFRP reinforcement was higher in all strokes. In addition, it is evident that B-pillar with CFRP has lesser bending deformation. The energies absorbed by B-pillar with DP590 and CFRP were 2.252 and 2.303 kJ, respectively. Therefore, the design rules proposed in this study were proven to be effective to replace steel products with CFRP products.

## Figures and Tables

**Table 1:** Mechanical properties of carbon fiber reinforced plastic (CFRP) laminate.

Mechanical Properties	Values
Elastic modulus in fiber direction (E_11_)	40.35 GPa
Elastic modulus in transverse direction (E_22_)	40.35 GPa
Shear modulus in 1–2 plane (G_12_)	9.51 GPa
Shear modulus in 2–3 plane (G_23_)	0.30 GPa
Shear modulus in 1–3 plane (G_13_)	0.30 GPa
Poisson’s ratio (ν_12_)	0.13

**Table 2:** Parameters of genetic algorithms (GAs).

Parameter	Value
Population size	100
Fiber array	0°, 15°, 30°, 45°, 60°, 75°
Probability of crossover	70%
Probability of mutation	5%
Crossover method	One-point crossover
Fitness evaluation	Tournament selection
